# Development and validation of a risk score model for predicting autism based on pre- and perinatal factors

**DOI:** 10.3389/fpsyt.2024.1291356

**Published:** 2024-02-16

**Authors:** Jianjun Ou, Huixi Dong, Si Dai, Yanting Hou, Ying Wang, Xiaozi Lu, Guanglei Xun, Kun Xia, Jingping Zhao, Yidong Shen

**Affiliations:** ^1^ Department of Psychiatry, and National Clinical Research Center for Mental Disorders, The Second Xiangya Hospital of Central South University, Changsha, Hunan, China; ^2^ Mental Health Center of Xiangya Hospital, Central South University, Changsha, Hunan, China; ^3^ Qingdao Mental Health Center, Qingdao, Shandong, China; ^4^ Shandong Mental Health Center, Jinan, Shandong, China; ^5^ Center for Medical Genetics and School of Life Sciences, Central South University, Changsha, Hunan, China

**Keywords:** autism spectrum disorder, risk factor, prediction, clinical screening, maternal environment

## Abstract

**Background:**

The use of pre- and perinatal risk factors as predictive factors may lower the age limit for reliable autism prediction. The objective of this study was to develop a clinical model based on these risk factors to predict autism.

**Methods:**

A stepwise logistic regression analysis was conducted to explore the relationships between 28 candidate risk factors and autism risk among 615 Han Chinese children with autism and 615 unrelated typically developing children. The significant factors were subsequently used to create a clinical risk score model. A chi-square automatic interaction detector (CHAID) decision tree was used to validate the selected predictors included in the model. The predictive performance of the model was evaluated by an independent cohort.

**Results:**

Five factors (pregnancy influenza-like illness, pregnancy stressors, maternal allergic/autoimmune disease, cesarean section, and hypoxia) were found to be significantly associated with autism risk. A receiver operating characteristic (ROC) curve indicated that the risk score model had good discrimination ability for autism, with an area under the curve (AUC) of 0.711 (95% CI=0.679-0.744); in the external validation cohort, the model showed slightly worse but overall similar predictive performance. Further subgroup analysis indicated that a higher risk score was associated with more behavioral problems. The risk score also exhibited robustness in a subgroup analysis of patients with mild autism.

**Conclusion:**

This risk score model could lower the age limit for autism prediction with good discrimination performance, and it has unique advantages in clinical application.

## 1 Introduction

Autism spectrum disorder (ASD, also referred to as autism) is a neurodevelopmental disorder characterized by deficits in social interaction and communication skills and by stereotyped behaviors and restricted interests (1). According to the most recent epidemiological study (2), the prevalence of ASD in the United States is 2.76%, and the lifetime cost is approximately $1.4 to 2.4 million (3). Early detection and intervention could improve the outcomes of patients with autism (4). The American Academy of Pediatrics (AAP) recommends that routine developmental screening tests be administered after 9 months of age, which can lead to further evaluation, diagnosis and intervention (5). In practice, however, early behavioral abnormalities may sometimes be neglected, and diagnoses are often delayed until the age of 4 or 5 years (6). Previous research has revealed that autism is highly heritable (7), and some autism-specific markers are present before 12 months of age or even at birth (8–10). These findings suggest that autism identification may be possible during the neonatal period. One of the causes of diagnostic delay may be a lack of efficiency in screening or diagnostic tools for very young individuals. Current tools are mostly based on behavioral factors rather than etiological or pathological factors, and behavioral signs of autism are not reliable before 12 months of age (11).

In addition to genetic factors, pre- and perinatal risk factors also play important roles in the etiology of autism (12, 13). Evidence has indicated that environmental risk factors, such as viral infection or exposure to toxins, can activate neuroinflammatory processes, increase oxidative stress and/or disturb signaling pathways, which may directly or indirectly affect the neurodevelopmental process and result in abnormal behaviors (14). Kim and colleagues conducted an umbrella review to systematically appraise relevant meta-analyses to explore the strength and validity of more than 60 environmental risk factors for autism. Several factors were found to be strongly associated with autism risk (e.g., maternal age older than 35 years, gestational hypertension, maternal overweight, pre-eclampsia and maternal antidepressant use) or had a high level of evidence (e.g., maternal age older than 30 years, high paternal age, maternal autoimmune disease, and acetaminophen use during pregnancy) (13). Although the physiopathological mechanism is unclear, utilizing these risk factors, which may be closely related to the etiology of autism, could lower the age limit for the prediction of autism. In addition, from a practical point of view, information on the above risk factors is relatively easy to obtain from parental reports or medical records and may be more convenient to apply in community settings, especially in rural or undeveloped areas. Hence, we conducted this case–control study to develop a clinical risk score model based on pre- and perinatal factors for predicting autism. In addition to its ability to predict autism, theoretically, this model can also be used for autism screening.

## 2 Methods

### 2.1 Subjects

The Human Ethics Committee of the Second Xiangya Hospital of Central South University reviewed and approved this study. There were two groups of participants in the study. The first group of participants was recruited from 2008 to 2016 to establish the model, and then, an independent cohort was recruited from 2018 to 2023 for external validation.

For the first sample, the participants in the autism group were recruited from the outpatient departments of three different hospitals in Hunan, Jiangsu and Sichuan Provinces and from four autism training centers in Hunan, Shandong and Henan Provinces. The birthplaces of these children were distributed among 28 provinces in China, and they were diagnosed with autism before recruitment. Participants in the control group were recruited from four kindergartens and a primary school in Hunan, Shandong and Jiangsu Provinces. After we obtained written informed consent from the children’s parents, two independent psychiatric doctors confirmed the diagnoses of the children in the autism group per the Diagnostic and Statistical Manual of Mental Disorders, 4^th^ edition, text revision (DSM-IV-TR) criteria for autism disorder. We designed a questionnaire to collect demographic information, medical history, and relevant information on risk factors during the prepregnancy, pregnancy, perinatal and postnatal periods from both groups. The parent-rated Social Responsiveness Scale (SRS) was used to evaluate children’s autistic traits. Parents of children above 4 years of age from both groups were also asked to complete the Achenbach Child Behavior Checklist (CBCL) to assess whether their children had any behavioral problems.

Children who presented with comorbid organic diseases of the nervous system were excluded from the study. Additionally, children in the control group were excluded if they had been previously diagnosed with psychiatric disorders or organic diseases of the nervous system or if their parents or teachers suspected a possible developmental disorder. Karyotype analysis performed at the Laboratory of Medical Genetics, Central South University, was used to identify participants in the autism group who had a chromosomal abnormality; these participants were also excluded from the study (n=12).

Ultimately, a total of 615 children with ASD and 615 children without ASD were recruited. All the recruited subjects were Han Chinese.

### 2.2 Candidate predictor selection and covariates

Data on candidate predictors were collected by adapting a parent-reported questionnaire. In total, 73 potential risk factors related to the prepregnancy, pregnancy, perinatal and postnatal periods were included in the questionnaire (**Supplementary Table 1**). The selection of these candidate predictors was based on two criteria. First, the percentage of missing data for each predictor had to be less than 10%; thus, the information was relatively reliable and could be effectively filled in. Second, the prevalence of the predictors had to be more than 1% in each group. For ease of interpretation and use, the continuous variables were categorized based on the medical reference range or previous literature. If the candidate variable had a U-shaped relationship with autism risk, the lowest risk was set as the reference. Candidate predictors that did not meet the prevalence criteria but showed an obvious increase in the incidence rate were merged into a new single variable for further analysis.

Ultimately, 28 candidate predictors (including 4 merged variables, shown in **Supplementary Table 2**) were selected for further analysis. Age at recruitment, sex, residential area and family annual income were included as covariates.

### 2.3 Statistical analysis

All the statistical analyses were conducted using IBM SPSS version 22.0. The chi-square test (Fisher’s exact test was used when necessary) was used to compare the selected variables between groups. Then, we filled in the missing values by adapting the expectation maximization (EM) algorithm. As all candidate predictors had missing data rates lower than 10%, the performance of the EM algorithm is similar to other filling methods such as multiple imputation (15).

After the missing values were filled and outliers were excluded, 80% of the participants were randomly selected into the training set, and the rest were included as the validation set. Then, a stepwise logistic regression analysis was conducted to explore the relationships between the selected predictors and autism risk in the training cohort (with recruitment age, residential area, and parental education levels included as covariates). The significance level of all the tests was set at p<0.05 (two-tailed). Nagelkerke R^2^ was used to assess the goodness of fit of the logistic model. The p values of multiple comparisons were corrected by Bonferroni correction.

We randomly split the data set into training and validation samples so that the performance of the model could be evaluated via cross-validation in the independent, unbiased validation sample. However, as data splitting may be less stable in smaller samples, we also conducted validation in our full sample using 1000 bootstrap replicates as a sensitivity analysis.

### 2.4 Risk score system development

To develop a practical scoring system, we assigned all the significant risk factors identified by the logistic model weights proportional to the β coefficient values (by dividing the β coefficients of each variable by the smallest coefficient in the model, multiplying by 2 and rounding to the nearest integer). The sum score for each participant in the training cohort was subsequently calculated. Student’s t test was used to compare the risk scores between the autism group and the control group. In addition, we constructed a linear regression model to analyze the association between the risk score and CBCL score.

A receiver operating characteristic (ROC) curve was generated to assess and compare the predictive accuracy of the risk score between different datasets. The maximal Youden index (sensitivity + specificity – 1) was used to determine the best cutoff point. The positive predictive value (PPV) and negative predictive value (NPV) were calculated based on the best cutoff point.

### 2.5 Model validation

#### 2.5.1 Validation of selected predictors

We input the 28 candidate risk factors into a chi-square automatic interaction detector (CHAID) decision tree for the training cohort for comparison with the logistic model in terms of the identified risk factors and the area under the ROC curve (AUC) values.We chose CHAID as an additional selection method because it enables a visual understanding of the selection process.

#### 2.5.2 Validation of the predictive performance

We adopted an established scoring system to calculate the risk score for the participants in the internal validation cohort. The AUC and the sensitivity, specificity, PPV and NPV of the internal validation cohort were compared with the results from the training cohort according to the same cutoff point.

#### 2.5.3 External independent validation

The external validation set included data from an independent clinical database that was developed from May 2018 to March 2023. The recruited participants included typically developing (TD) and children with ASD as well as children with other neurodevelopmental disorders (ONDs). The pre- and perinatal information was partly obtained from parental reports via online questionnaires, and the rest of the information was obtained from a clinical interview at the time of recruitment. Because the questionnaires used in the two studies were different, we first matched the items carefully. The matched items are presented in **Supplementary Table 3**.

The completion rate of the online questionnaires was 84.5%. After excluding subjects and outliers from the dataset, we identified 614 children with ASD (513 males, 101 females), 115 TD children (68 males, 57 females), and 98 children with ONDs (75 males, 23 females). The mean recruitment age of the children with autism was 4.38 years (SD=1.53 years), the mean recruitment age of the TD children was 6.35 years (SD=2.62 years), and the mean recruitment age of the children with ONDs was 7.64 years (SD=2.71 years). Diagnoses were based on the DSM-5 criteria, and relevant developmental and medical history information was also checked. In addition, the Aberrant Behavior Checklist (ABC) (16) was used to assess the severity of behavioral symptoms in this sample. The same statistical methods used for internal validation of the model were applied for external validation.

### 2.6 Performance of the risk score model in the mild autism subgroup

We analyzed the performance of the risk score model in the subgroup of children with mild autism to test its robustness. In the present study, participants in the mild autism subgroup were defined as those whose SRS total raw score was less than 65. The SRS is widely used to measure autistic traits in the general population, and it performs extraordinarily well in autism screening (17). However, similar to other parent-report screening tools, the SRS is a subjective rating scale, and its performance in screening is reduced when the child’s social impairment is not prominent or when the rater lacks sufficient awareness of autistic symptoms. Our current predictive model based on prenatal and perinatal risk factors may be robust and more suitable for the above conditions. Therefore, we compared the performance of the risk score model and the SRS among the subgroup of children with mild autism. The analyses were subsequently used to calculate the theoretical sensitivity and specificity of the risk score model combined with the SRS to form a serial screening test or a parallel screening test, which can extend the usage of our risk score model to specific clinical subgroups or conditions.

## 3 Results

### 3.1 Characteristics and comparison of selected predictors and participants

As shown in **Table 1**, the mean age of the participants in the case group was 5.01 years (SD=1.25 years), ranging from 2.33 to 10.38 years. The mean age of the participants in the control group was 4.76 years (SD=1.32 years), ranging from 2.12 to 13.35 years. Significant differences were found in 8 of the 28 candidate predictors after Bonferroni correction for multiple comparisons (details shown in **Supplementary Table 2**).

**Table 1:** Characteristics and comparisons of recruited samples.

		Case	Control			t/χ^2^	p value		
1. Whole sample (615 cases, 615 controls)									
Age of children(years, mean±SD)		5.01±1.25	4.76±1.32			3.49	0.001	
Sex, n (%)								
Male		525(85)	312(50.7)			169.65	<0.001		
Female		90(14.6)	303(49.3)						
Family annual income(RMB), n(%)									
Less than 50,000		272(44.2)	177(28.8)			53.23	<0.001		
50,000-100,000		225(36.6)	214(34.8)						
More than 100,000		118(19.2)	224(36.4)						
Living Area, n (%)								
Urban		439(71.4)	389(63.3)			85.67	<0.001		
Suburb		121(19.7)	56(9.2)						
Rural areas		55(8.9)	170(27.6)						
2. Training Set (492 cases, 492 controls)									
Age of children(years, mean±SD)		4.98±1.23	4.78±1.39			2.37	0.02		
Sex, n (%)								
Male		423(86.0)	250(50.8)			140.77	<0.001		
Female		69(14.0)	242(49.2)					
Family annual income(RMB), n(%)									
Less than 50,000		222(45.1)	133(27.0)			49.22	<0.001		
50,000-100,000		178(36.2)	182(37.0)						
More than 100,000		92(18.7)	177(36.0)						
Living areas, n (%)									
Urban		353(71.7)	318(64.6)			68.02	<0.001		
Suburb		95(19.4)	41(8.3)						
Rural areas		44(8.9)	133(27.0)						
3. Validation Set (123 cases, 123 controls)									
Age of children(years, mean±SD)		5.17±1.32	4.68±0.98			3.28	0.001	
Sex, n (%)									
Male		102(82.9)	62(50.4)			29.27	<0.001		
Female		21(17.1)	61(49.6)						
Family annual income(RMB), n(%)									
Less than 50,000		50(40.7)	44(35.8)			9.27	0.01		
50,000-100,000		47(38.2)	32(26.0)						
More than 100,000		26(21.1)	47(38.2)						
Living areas, n (%)									
Urban		86(69.9)	71(57.7)			18.47	<0.001		
Suburb		26(21.2)	15(12.2)						
Rural areas		11(8.9)	37(30.1)						

### 3.2 Potential risk factors for autism

As shown in **Table 2**, forward stepwise logistic regression identified five predictors associated with the risk of autism. In addition to the covariates, the logistic model included three predictors from the pregnancy period (influenza-like illness, stress and maternal allergic/autoimmune disease) and two predictors from the perinatal and neonatal periods (cesarean section and hypoxia). These five selected factors explained a fair amount of the variation in ASD risk (the Nagelkerke R^2^ of the final model was 0.450).

**Table 2:** Identified risk factors for autism.

	β	OR(95%CI)	p	Score ^#^
1. Pregnancy Influenza-like illness	0.88	2.40(1.57-3.68)	<0.001	4
2. Caesarean section	0.46	1.56(1.16-2.17)	0.004	2
3. hypoxia ^a^	1.31	3.70(1.97-6.95)	<0.001	6
4. Maternal allergic/auto-immune disease ^b^	0.89	2.44(1.39-4.26)	0.002	4
5. Pregnancy stressor ^c^	0.89	2.43(1.62-3.66)	<0.001	4

a Merged from three predictors included Perinatal intrauterine asphyxia, Neonatal asphyxia caused by meconium aspiration or other conditions, and Neonatal respiratory distress.

b Merged from Maternal allergic diseases (allergic dermatitis/allergic rhinitis/allergic asthma/allergic purpura/allergic shock), and Maternal auto-immune diseases(SLE/rheumatoid arthritis/eczema/asthma).

c Merged from three relative predictors which included Pregnancy psychological trauma, Severe family conflicts during pregnancy, and Persistent emotional problems during pregnancy (anxiety or depression).

^#^ Calculated by dividing the β coefficients of each variable by the smallest coefficient in the model, multiplying by 2 and rounding to the nearest integer.

### 3.3 Risk scores for autism prediction

**Figure 1:**
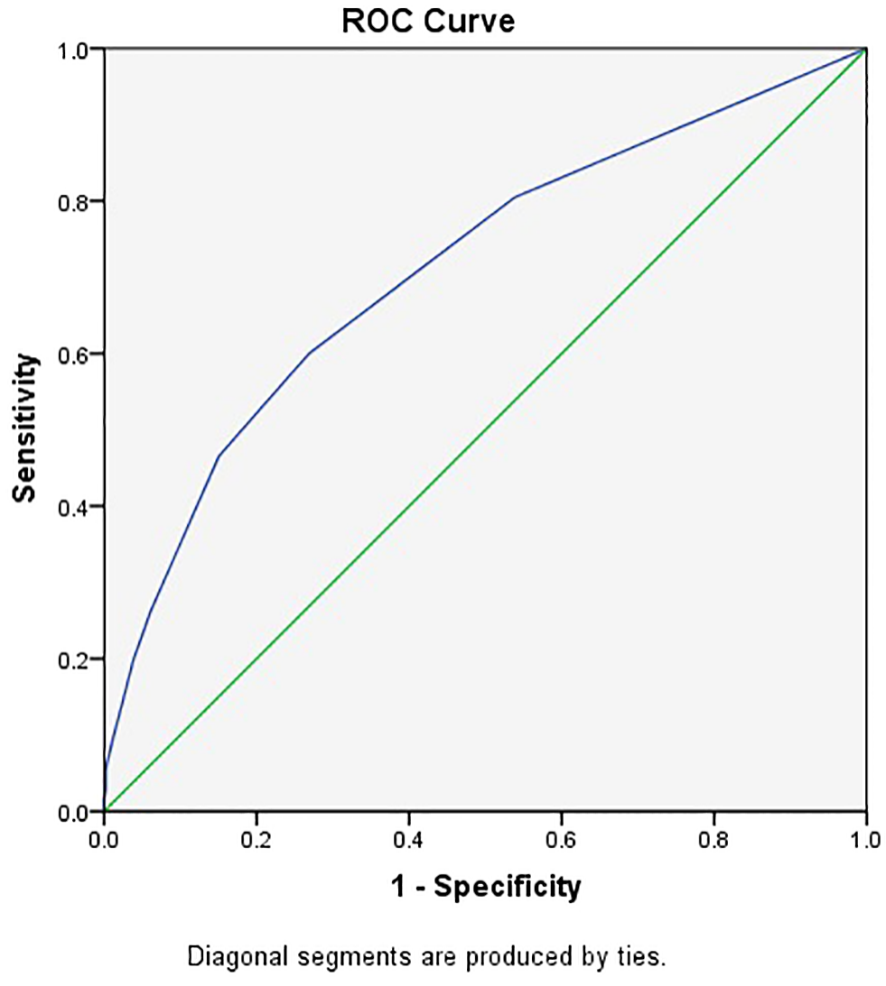
ROC curve for clinical score of autism.

The weighted scores of each identified risk factor are shown in **Table 2**. The minimum sum score of the scoring system was 0, and the maximum score was 20. In the training set, the sum score ranged from 0 to 20 in the autism group and from 0 to 16 in the control group. Student’s t test revealed a significant difference between the groups (mean ± SD: 5.08 ± 4.39 for the autism group vs. 2.15 ± 2.77 for the control group, t=12.53, p<0.001). The risk score system showed good discrimination for autism, with an AUC of 0.711 (95% CI=0.679-0.744) in the training cohort (**Figure 1**). The best estimated cutoff score was 3. With this cutoff score, the sensitivity was 0.600, the specificity was 0.732, the NPV was 0.646, and the PPV was 0.691.

### 3.4 Model validation

**Figure 2:**
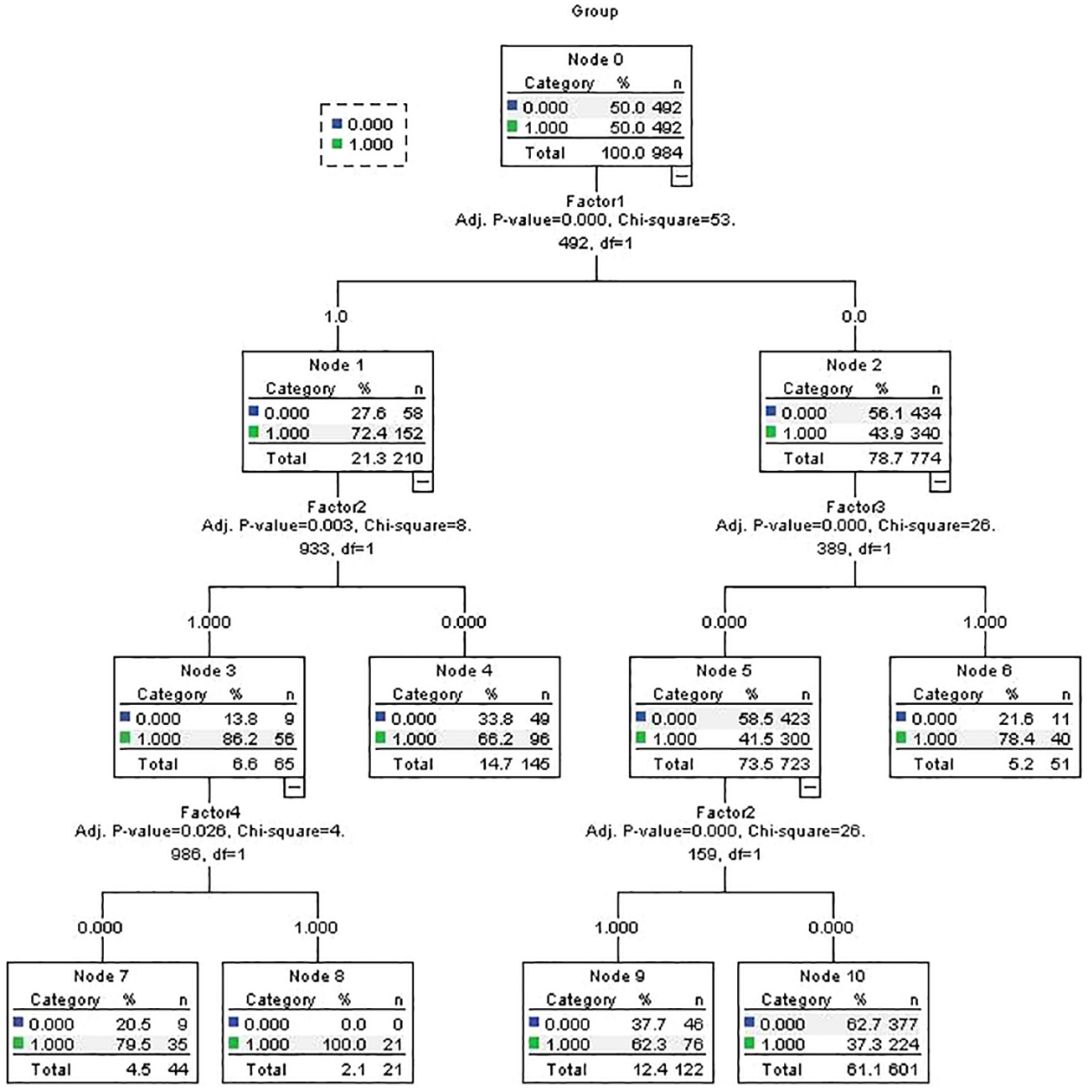
CHAID decision tree predicting autism diagnosis related factors in training samples. Factor 1= Pregnancy stressor; Factor 2= Pregnancy Influenza-like illness; Factor 3= Hypoxia; Factor 4= Maternal allergic/Auto-immune disease;.

We input the 28 candidate risk factors into a CHAID decision tree in the same training cohort for comparison with the logistic model. The logistic regression results revealed that four of the five selected factors (influenza-like illness during pregnancy, hypoxia, maternal allergic/autoimmune disease and stress during pregnancy) were also included in the decision tree model as key factors (**Figure 2**). The predictive AUC value of the decision tree was 0.670 (95% CI=0.636-0.703).

The risk score for the validation cohort was calculated; the mean score of the ASD group was 4.98 ± 4.27, and that of the control group was 2.31 ± 2.84. A significant difference was detected between the groups (t=5.77, p<0.001). The AUC was 0.700 (95% CI: 0.635-0.765). This result was very similar to that of the training cohort. By adapting the same cutoff score, the sensitivity was 0.602, the specificity was 0.691, the NPV was 0.634, and the PPV was 0.661. Comparisons of the predictive performance among the samples are shown in **Table 3**. The similarity of the results indicates that the risk score system performed well in both the training and internal validation cohorts. Then, we combined the training and internal validation cohorts to calculate the predictive performance for all participants. The AUC was 0.709 (95% CI=0.681-0.738). With respect to the best cutoff score, the sensitivity was 0.600, the specificity was 0.724, the NPV was 0.644, and the PPV was 0.685. The 95% CI of AUC using 1000 bootstrap replicates from the full sample was 0.662-0.715.

**Table 3:** Comparisons of predictive performance of different samples.

Model	AUC(95%CI)	Predictive performance of best cut-off point ^a^			
		Sensitivity	Specificity	PPV	NPV
Training sample	0.711 (0.679-0.744)	0.600	0.732	0.691	0.646
Internal validation sample	0.700 (0.635-0.765)	0.602	0.691	0.661	0.634
Whole sample	0.709 (0.681-0.738)	0.600	0.724	0.685	0.644

a The best cutoff point 3 was determined the maximal Youden index.

In the independent validation cohort, the mean (SD) scores of the ASD group, TD group, and OND group were 4.55 (3.96), 1.89 (2.14) and 3.21 (2.84), respectively. *Post hoc* analysis revealed significant differences in the mean scores between the ASD group and the TD group (p<0.001) as well as between the OND group and the TD group (p=0.007). A significant difference was also found in the mean risk score between the ASD and OND groups (p=0.001). The predictive AUC of the independent validation cohort was 0.657 (95% CI=0.618-0.697). With the same cutoff point (3 points), the sensitivity was 0.570, and the specificity was 0.714. Thus, the risk score system did not perform as well as it did in the original training cohort, but its performance was still acceptable. A comparison of the ROC curves of the different cohorts is shown in **Supplementary Figure 1**.

The calibration plot of different sample set showed good calibration for the prediction model(see **Supplementary Figures 2-4**).

### 3.5 Robustness of the risk score model in the mild autism subgroup

Before comparing the diagnostic performance of the risk score model with that of the SRS in the mild subgroup, we first verified the ability of the SRS to distinguish ASD patients from TD children in the whole sample. The results showed that at its best cutoff point, the AUC for the SRS was 0.932 (95% CI: 0.917-0.946), the sensitivity was 0.852, and the specificity was 0.900, which was consistent with previous studies (18). When only children with autism with SRS total raw scores less than 65 were included in the analysis, the AUC, sensitivity and specificity decreased to 0.732, 0.662 and 0.743, respectively. The performance of the risk score model remained stable among the subgroups, and the AUC, sensitivity and specificity were 0.709, 0.574 and 0.731, respectively. The results revealed the robustness of the risk model in children with different clinical conditions. Moreover, theoretically, when the risk score model and the SRS are combined to form a serial screening test, the sensitivity for screening the mild autism subgroups may increase to 0.856; when combined as a parallel screening test, it may increase specificity to 0.928. Taken together, these findings indicate that the combination of the risk score model with the SRS (or other screening tools) may extend its usage and improve screening efficiency in mild autism subgroups.

### 3.6 Association between risk scores and behavioral problems in children

The parent-completed CBCL was used to assess the behavioral problems of children older than 4 years. We constructed a linear regression model to analyze the association between the risk score and CBCL score, as shown in **Supplementary Table 4**. The risk score was significantly positively associated with the CBCL total raw score in both groups after adjusting for recruitment age and sex (all p<0.05); thus, a higher risk score was associated with more behavioral problems.

## 4 Discussion

Using multivariable logistic regression, we found that several environmental risk factors during the pre- and perinatal periods are associated with autism risk. These risk factors include influenza-like illness during pregnancy, stress during pregnancy, maternal allergic/autoimmune disease, cesarean section, and hypoxia. Based on these identified factors, we developed a model for predicting autism risk. We used a CHAID tree as a second statistical tool and an external validation cohort and found that the developed risk score model exhibited satisfactory predictive performance. To our knowledge, this study has the largest sample size among similar studies in the Chinese population and is the first to develop a predictive model based on prenatal and perinatal risk factors.

Associations between the five identified risk factors and autism have been reported by several previous studies, including high-quality meta-analyses and large-sample studies (19–25). However, other studies failed to find significant associations between these factors (26–28). These inconsistencies may be caused by differences in methodology (including the study design, diagnostic criteria, sample size and data sources) rather than differences in pathophysiology mechanisms (29). For example, regarding influenza-like illness during pregnancy, a large population-based cohort study conducted in Denmark compared maternal-reported data and hospital data and revealed that the agreement between maternal-reported infection and hospital-registered infection was good for some specific infections (cystitis, 66%; vaginal yeast infection, 77%), but poor for respiratory infection and influenza (6% and 7%, respectively) (19). This finding suggested that data from medical records may be more precise but less sensitive to subclinical infection and illness for mothers who do not seek medical help (30). In the same study mentioned above, based on self-reported information, the results indicated that influenza-like infection during pregnancy was associated with a twofold increased risk of autism (adjusted hazard ratio [aHR]=2.3, 95% CI=1.0-5.3). In addition to selection bias, there is still no national medical registry system available in China (and thus a lack of complete medical records for all subjects); therefore, we collected data from parental reports only.

One of the barriers in translating environmental risk factors into a predictive model is that any single factor or event has only a limited contribution to the etiology and is unlikely to cause autism in isolation (31). Furthermore, in some of the previous studies, only univariate analyses were performed, and confounders were not properly assessed (29). Several researchers have suggested that many risk factors may be associated with a common pathway (32). We combined a broad, comprehensive survey with multivariable statistical methods, providing an advantage in the exploration of potential “biological interactions” among risk factors and the selection of the strongest factors when developing the prediction model. A subsequent concern may arise that there is overlap among the selected factors in all ONDs. However, we think that the contributions of these factors could vary according to the disorder; even though some factors seem common intuitively, statistical results reveal their specificity for a specific disorder. By combining these factors and considering different weights and their interactions, it was possible to construct a prediction model that met the specificity requirements.

The second strength of our study is that we used independent data (i.e., the external validation cohort) to validate the developed model. The predictive performance in the external validation cohort was slightly worse than that in the original sample, possibly because some items were not well matched. However, similar results were achieved in an independent cohort with a different design and a different source, indicating that the findings are robust and that the simple risk score is valid and has clinical utility. Moreover, the independent cohort included subjects with ONDs, and comparison revealed that the mean score of the ASD group was significantly greater than that of the OND group. Thus, to a certain extent, these results could help to answer some potential questions related to shared risk factors when this model is applied in clinical settings.

The sensitivity and specificity of our model were acceptable but not outstanding; therefore, we do not propose that the current model be used to provide a precise estimate of autism risk in clinical practice. Nevertheless, as there is currently no standardized screening or prediction tool appropriate for use in all children or in children of all ages (and as shown by our analysis), combining the current model with other tests may be a reasonable way to increase the efficiency of ASD screening or distinguish ASD from ONDs. On the other hand, as described in the recommendations for early screening of ASD (11), lack of time, unfamiliarity with screening instruments, or difficulty in scoring among providers are potential barriers to early screening of ASD. Our model uses prenatal and perinatal risk information, which is easy and convenient to collect and may be more suitable for use in economically underdeveloped regions. In addition, when combined with developmental trajectories, the risk score model may help nonspecialist pediatricians or primary care physicians identify the children most in need of routine developmental surveillance.

In addition to clinical applications, the present study and the risk score model may also stimulate research on environmental risk factors in clinical settings. The significant correlation between CBCL and the risk score, reflects more adverse events in prenatal or perinatal period may cause more behaviors problems in offspring (33). Meanwhile, the results also suggests that our risk score may closely align with the underlying physiopathological mechanism, even though it is not currently feasible to directly assess behavioral problems by using the risk score.

However, there are several limitations of our study. First, the sample size was relatively small, especially for some factors with a low incidence, which may have led to insufficient statistical power and relatively less stability by adopting data splitting for internal validation. Hence, a larger sample size is needed in future studies. Second, when collecting the risk factor data, both the testing and validation cohorts were recruited with a retrospective design, which may contribute to the possibility of recall bias (although several previous studies have indicated that parental reports are largely consistent with contemporaneous medical records for some pregnancy events in neurodevelopmental disorder research (34)). Therefore, a study with a prospective design or the use of medical records would help to confirm the findings. Third, we did not perform a detailed characterization of the children with autism. Most notably, we did not measure intelligence quotients; one may expect that pre- or perinatal risk factors are much more common in individuals with moderate to severe intellectual disability. However, this information was not available for the current sample of children with autism; thus, model extrapolation may be impacted. Finally, detailed information about exposure duration and intensity was not available in the study, and exposure duration and intensity may have affected the outcome (35). However, the effects of these variables are uncertain (36). We also think that an overly detailed model may have lower clinical utility.

As the proposed model is not a final model but rather a preliminary framework that can be modified, it can be improved by research with a larger sample size and broader and more detailed information combined with other screening measurements, biomarkers or genotyping data. In conclusion, we developed a clinical predictive model based on five identified prenatal and perinatal risk factors for autism that can effectively predict or screen for autism. The model was validated by the use of a second statistical method and an external independent cohort. This risk score model could lower the age limit for autism prediction and screening to the first month after birth.

## Data availability statement

The raw data supporting the conclusions of this article will bemade available by the authors, without undue reservation.

## Ethics statement

The studies involving humans were approved by the Human Ethics Committee of the Second Xiangya Hospital of Central South University. The studies were conducted in accordance with the local legislation and institutional requirements. Written informed consent for participation in this study was provided by the participants’ legal guardians/next of kin.

## Author contributions

JO: Formal Analysis, Data curation, Validation, Writing – original draft. HD: Data curation, Formal Analysis, Validation, Writing – original draft. SD: Data curation, Investigation, Writing – review & editing. YH: Data curation, Investigation, Writing – review & editing. YW: Data curation, Investigation, Writing – review & editing. XL: Data curation, Investigation, Writing – review & editing. GX: Data curation, Investigation, Writing – review & editing. KX: Supervision, Writing – review & editing. JZ: Supervision, Writing – review & editing. YS: Conceptualization, Formal Analysis, Methodology, Writing – review & editing.

## Supplementary material

SUPPLEMENTARY FIGURE 1 Comparison of ROC curves from different sample sets.

Supplementary Figure 2 The calibration plot of the training sample set.

Supplementary Figure 3 The calibration plot of the internal validation sample set.

Supplementary Figure 4 The calibration plot of the independent sample set.
