# Supplementary material for: Development and validation of a risk score model for predicting autism based on pre- and perinatal factors
Source: Front Psychiatry. 2024 Feb 16;15:1291356. doi: 10.3389/fpsyt.2024.1291356 (PMC10904522; doi:10.3389/fpsyt.2024.1291356)
Supplement: Supplementary file 1 [file DataSheet_1.docx]

Table S1. List of items included in the parents’ questionnaire.

| Item | Data type/Coded |
| --- | --- |
| **General information** | |
| Age | continuous |
| Gender | binary |
| Family annual income | ordinal categorical  1= <50000 Yuan  2=50000-100000 Yuan  3= >100000 Yuan |
| Education levels of parents | ordinal categorical  1= primary school  2= junior high school  3= senior high school  4= college  5= higher levels |
| **Pre-pregnancy** | |
| Maternal age at delivery^✝^ | continuous |
| Paternal age at delivery^✝^ | continuous |
| Use of tobacco before pregnancy | binary |
| Use of alcohol before pregnancy | binary |
| Use of drugs before pregnancy | binary |
| Maternal allergic disease(allergic dermatitis/ allergic rhinitis/ allergic asthma/ allergic purpura/ allergic shock) | binary |
| Paternal allergic disease(allergic dermatitis/ allergic rhinitis/ allergic asthma/ allergic purpura/ allergic shock) | binary |
| Maternal working environment before pregnancy is safety and health | binary |
| Maternal working environment before pregnancy is easy to exposure to bacteria and virus before pregnancy | binary |
| Maternal working environment before pregnancy is dirty and humid | binary |
| Maternal working environment before pregnancy is easy to exposure to toxic substances | binary |
| Paternal working environment is safety and health | binary |
| Paternal working environment is easy to exposure to bacteria and virus before pregnancy | binary |
| Paternal working environment is safety and health | binary |
| Paternal working environment is easy to exposure to toxic substances | binary |
| Maternal Diseases before pregnancy | binary |
| Paternal Diseases before pregnancy | binary |
| Maternal body mass index before pregnancy^✝^ | continuous |
| Mothers prepared before pregnancy (supplement with folic acid/vitamins) | binary |
| Abortion history | ordinal categorical  1=1  2=2  3= more than 2 |
| Parity^✝^ | continuous |
| **Pregnancy** |  |
| Became pregnant while taking birth control pills | binary |
| Threatened abortion | binary |
| Threatened premature | binary |
| Malposition | binary |
| Nuchal encirclements of umbilical cord | binary |
| RH blood group incompatibility during pregnancy | binary |
| Abnormal thyroid function during pregnancy | binary |
| Autoimmune diseases during pregnancy (SLE; rheumatoid arthritis; eczema; asthma) | binary |
| Severe trauma during pregnancy | binary |
| Gestational diabetes mellitus | binary |
| Severe pregnancy reaction | binary |
| Pregnant Influenza-like illness | binary |
| Ascending infection | binary |
| Kidney infection | binary |
| Other infection (RV/CMV/HSV/TOX) during pregnancy | binary |
| Pregnancy hypertension (PIH) | binary |
| Pregnancy edema | binary |
| Eclampsia | binary |
| Placenta previa/ placental abruption | binary |
| Persistent emotional problems during pregnancy (pregnancy anxiety; pregnancy depression) | binary |
| Use of tobacco during pregnancy | binary |
| Use of alcohol during pregnancy | binary |
| Use of drugs during pregnancy | binary |
| Use of medicine during pregnancy | binary |
| Psychological trauma during pregnancy | binary |
| Severe family conflicts during pregnancy | binary |
| Gestational weight gain^☨^ | continuous |
| Pregnancy poisoning | binary |
| **Perinatal period** | |
| Labor process | categorical  1= precipitate labor  2= normal delivery  3= prolonged labor |
| Mode of delivery | categorical  1= premature birth  2= postmature birth  3= full-term birth |
| Premature rupture of membranes | binary |
| Polyhydramnios | binary |
| Oligohydramnios | binary |
| Caesarean section | binary |
| Reason for cesarean section | categorical  1= vaginal delivery may be difficult, chose cesarean before delivery  2= vaginal delivery is difficult, chose cesarean during delivery  3= initiative selection |
| Birth length^✝^ | continuous |
| Birth Weight^✝^ | continuous |
| Use of oxytocin | binary |
| Delivery of anesthesia (epidural anesthesia) | binary |
| Delivery of anesthesia (general anesthesia) | binary |
| Dystocia at birth | binary |
| Asphyxia at birth | binary |
| Fetal bradycardia at birth | binary |
| Breech presentation | binary |
| Meconium aspiration | binary |
| Neonatal asphyxia | binary |
| Neonatal respiratory distress | binary |
| Neonatal severe infection | binary |
| Neonatal pathological jaundice | binary |
| Neonatal scleroderma | binary |
| Neonatal anemia | binary |
| Neonatal convulsions with unclear causes | binary |

^✝^ The continuous variables were categorized as follows: Maternal at delivery (1= <30 years old; 2= ≥30 years old), Paternal at delivery (1= <30 years old; 2= ≥30 years old), Maternal body mass index before pregnancy(1= <18.5 kg/m^2^; 2=18.5 kg/m^2^-24.0 kg/m^2^;3= >24.0 kg/m^2^), Birth Weight (1= <2.5kg; 2=2.5-4kg; 3= >4kg), Birth length (1= <48cm, 2=48-52cm, 3= >52cm), Parity (1=1; 2=2; 3=3; 4= ≥4).

^☨^Categorizing for gestational weight gain was based on the maternal body mass index: [1= Inadequate(<15kg for lean individuals, <13 kg for normal individuals, <10 kg for overweight/obese individuals); 2= adequate(15–22 kg for lean individuals, 13–21 kg for normal individuals,10–18 kg for overweight/obese individuals); 3=excessive(>22kg for lean individuals, >21 kg for normal individuals, >18 kg for overweight/obese individuals)]

Table S2 Comparison of selected candidate predictors in training set

|  | | Case | Control | | | χ^2^ | p value | |
| --- | --- | --- | --- | --- | --- | --- | --- | --- |
| **1. Pre-pregnancy & pregnancy** | | | | | |  |  | |
| Maternal age at delivery, n (%) | |  |  | | |  |  | |
| ≥30 years old | | 120(24) | 121(25) | | | 0.06 | 0.94 | |
| <30 years old | | 372(76) | 371(75) | | |  |  | |
| Paternal age at delivery, n (%) | |  |  | | |  |  | |
| ≥30 years old | | 239(49) | 254(52) | | | 0.92 | 0.34 | |
| <30 years old | | 253(51) | 238(48) | | |  |  | |
| Pregnancy stressor ^a^,n (%) | | | | | | | | |
| Yes | 152(31) | | | 58(12) | 53.49 | | | **<0.001** |
| No | 340(69) | | | 434(88) |  |  |  |  |
| Maternal allergic and auto-immune diseases ^b^, n (%) | | | | | | | | |
| Yes | | 84(17) | 25(5.1) | | | 35.91 | **<0.001** | |
| No | | 408(83) | 467(94.9) | | |  |  |  |
| Paternal allergic disease, n (%) | | | | | | | | |
| Yes | | 61(12) | 30(6.1) | | | 11.64 | **0.001** | |
| No | | 431(88) | 462(93.9) | | |  |  |  |
| Became pregnant while taking birth control pills, n (%) | | | | | |  |  | |
| Yes | | 11(2.2) | 12(2.4) | | | 0.05 | 0.83 | |
| No | | 481(97.8) | 480(97.6) | | |  |  |  |
| Vaginal bleeding ^c^, n (%) | | | | | |  |  | |
| Yes | | 65(13) | 36(7.3) | | | 9.28 | 0.002 | |
| No | | 427(87) | 456(92.7) | | |  |  |  |
| Polyhydramnios, n (%) | | | | | | | | |
| Yes | | 29(5.9) | 25(5.1) | | | 0.31 | 0.58 | |
| No | | 463(94.1) | 467(94.9) | | |  |  |  |
| Threatened premature labor, n (%) | | | | | |  |  | |
| Yes | | 32(6.5) | 16(3.3) | | | 5.61 | 0.02 | |
| No | | 460(93.5) | 476(96.7) | | |  |  |  |
| Nuchal cord, n (%) | | | | | |  |  | |
| Yes | | 179(36) | 105(21) | | | 27.11 | **<0.001** | |
| No | | 313(64) | 387(79) | | |  |  |  |
| RH blood group incompatibility during pregnancy, n (%) | | | | | |  |  | |
| Yes | | 8(1.6) | 13(2.6) | | | 1.22 | 0.27 | |
| No | | 484(98.4) | 479(97.4) | | |  |  |  |
| Gestational diabetes mellitus (GDM), n (%) | | | | | |  |  | |
| Yes | | 19(3.9) | 8(1.6) | | | 4.61 | 0.03 | |
| No | | 473(96.1) | 484(98.4) | | |  |  |  |
| Severe pregnancy reaction, n (%) | | | | | |  |  | |
| Yes | | 115(23) | 103(21) | | | 0.85 | 0.36 | |
| No | | 377(77) | 389(79) | | |  |  |  |
| Pregnancy Influenza-like illness (ILI), n (%) | | | | | |  |  | |
| Yes | | 141(29) | 57(12) | | | 44.61 | **<0.001** | |
| No | | 351(71) | 435(88) | | |  |  |  |
| Ascending infection, n (%) | | | | | |  |  | |
| Yes | | 38(7.7) | 18(3.7) | | | 7.57 | 0.006 | |
| No | | 454(92.3) | 474(96.3) | | |  |  |  |
| Other infection (RV/CMV/HSV/TOX) during pregnancy, n (%) | | | | | | | | |
| Yes | | 15(3.0) | 6(1.2) | | | 3.94 | 0.05 | |
| No | | 477(97.0) | 486(98.8) | | |  |  |  |
| Pregnancy hypertension (PIH), n (%) | | | | | |  |  | |
| Yes | | 16(3.3) | 7(1.4) | | | 3.61 | 0.06 | |
| No | | 476(96.7) | 485(98.6) | | |  |  |  |
| Smoking/passive smoking during pregnancy, n (%) | | | | | |  |  | |
| Yes | | 110(22) | 72(15) | | | 9.74 | 0.002 | |
| No | | 382(78) | 420(85) | | |  |  |  |
| **2. Perinatal & neonatal period** | | | | | |  |  | |
| Mode of delivery, n (%) | | | | | |  |  | |
| 1= premature birth | | 33(6.7) | 16(3.3) | | | 10.67 | 0.005 | |
| 2= postmature birth | | 29(5.9) | 16(3.3) | | |  |  |  |
| 3= full-term birth | | 430(87.4) | 460(93.4) | | |  |  |  |
| Dystocia at birth, n (%) | | | | | |  |  | |
| Yes | | 22(4.5) | 13(2.6) | | | 2.40 | 0.12 | |
| No | | 470(95.5) | 479(97.4) | | |  |  |  |
| Caesarean section, n (%) | | | | | |  |  | |
| Yes | | 286(58) | 194(39) | | | 34.43 | **<0.001** | |
| No | | 206(42) | 298(61) | | |  |  |  |
| Use of oxytocin, n (%) | | | | | |  |  | |
| Yes | | 105(21) | 92(19) | | | 1.07 | 0.30 | |
| No | | 387(79) | 400(81) | | |  |  |  |
| Anesthesia (epidural anesthesia/ general anesthesia), n (%) | | | | | |  |  | |
| Yes | | 306(62) | 245(50) | | | 15.35 | **<0.001** | |
| No | | 186(38) | 247(50) | | |  |  |  |
| Fetal bradycardia at birth, n (%) | | | | | |  |  | |
| Yes | | 32(6.5) | 16(3.3) | | | 5.61 | 0.02 | |
| No | | 460 (93.5) | 476(96.7) | | |  |  |  |
| Breech presentation, n (%) | | | | | |  |  | |
| Yes | | 17(3.5) | 21(4.3) | | | 0.44 | 0.51 | |
| No | | 475(96.5) | 471(95.7) | | |  |  |  |
| Neonatal pathological jaundice, n (%) | | | | | |  |  | |
| Yes | | 67(14) | 47(9.6) | | | 3.97 | 0.05 | |
| No | | 425(86) | 445(90.4) | | |  |  |  |
| Neonatal anemia, n (%) | | | | | |  |  | |
| Yes | | 5(1.0) | 8(1.6) | | | 0.70 | 0.40 | |
| No | | 487(99.0) | 484(98.4) | | |  |  |  |
| hypoxia ^d^ | | | | | | | | |
| Yes | | 70(14) | 18(3.7) | | | 33.75 | **<0.001** | |
| No | | 422 (86) | 474 (96.3) | | |  |  | |

^a^ Merged from three relative predictors which included Pregnancy psychological trauma, Severe family conflicts during pregnancy, and Persistent emotional problems during pregnancy (anxiety or depression).

^b^ Merged from Maternal allergic diseases (allergic dermatitis/allergic rhinitis/allergic asthma/allergic purpura/allergic shock), and Maternal auto-immune diseases (SLE/rheumatoid arthritis/eczema/asthma).

^c^ Merged from Threatened abortion and Placenta previa/ placental abruption.

^d^ Merged from three predictors included Perinatal intrauterine asphyxia, Neonatal asphyxia caused by meconium aspiration or other conditions, and Neonatal respiratory distress.

Significant differences after the Bonferroni adjustment were showed in bold.

Table S3 The matched items from two questionnaire

| **The items for Chinese samples** | **The matched items for external validation samples** |
| --- | --- |
| A. Pregnancy Influenza-like illness | a1. Viral or Flu-like illness  a2. Viral illness(cold/influenza)  a3. Low grade fever  a4. Fever of 101 or above |
| B. Stressor during pregnancy | b1. Antidepressant/depression/anxiety |
| C. Maternal allergic/auto-immune disease | c1. Asthma  c2. Severe allergies requiring medication treatment  c3.Medications for asthma  c4. Anti-inflammatory or anti-immune drugs |
| D. Caesarean section | d1. Caesarean |
| E. hypoxia | e1. Difficulties in getting child to breathe on his/her own |
|  | e2. Umbilical cord around baby's neck |
|  | e3. Did this child need to have resuscitation, such as, having the nurses and doctors help him/her breathe or get his/her heart started in the delivery room? |

Table S4. The association of the risk score and CBCL total raw score^a^

| **Group** | **β** | **t** | **p** |
| --- | --- | --- | --- |
| TD group(n=445) | 0.23 | 4.96 | <0.001 |
| Autism group(n=482) | 0.13 | 2.77 | 0.006 |
| Whole subjects(n=927) | 0.24 | 7.46 | <0.001 |

^a^ CBCL total raw score as the dependent variable. Model adjusted by sex and recruited age.
