# Supplementary figures and images for: Development and validation of a risk score model for predicting autism based on pre- and perinatal factors
Source: Front Psychiatry. 2024 Feb 16;15:1291356. doi: 10.3389/fpsyt.2024.1291356 (PMC10904522; doi:10.3389/fpsyt.2024.1291356)

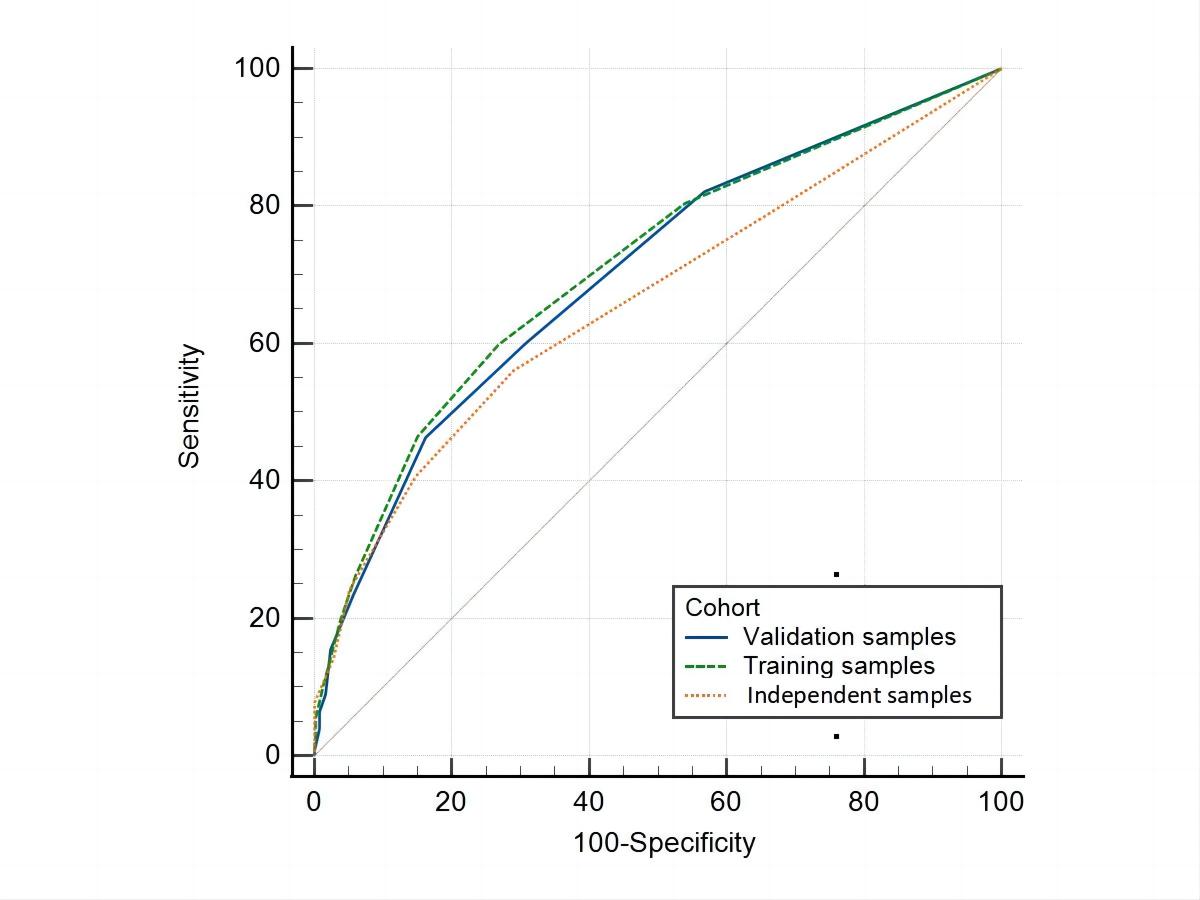

Supplement: SUPPLEMENTARY FIGURE 1 — Comparison of ROC curves from different sample sets. [file Image_1.jpeg]

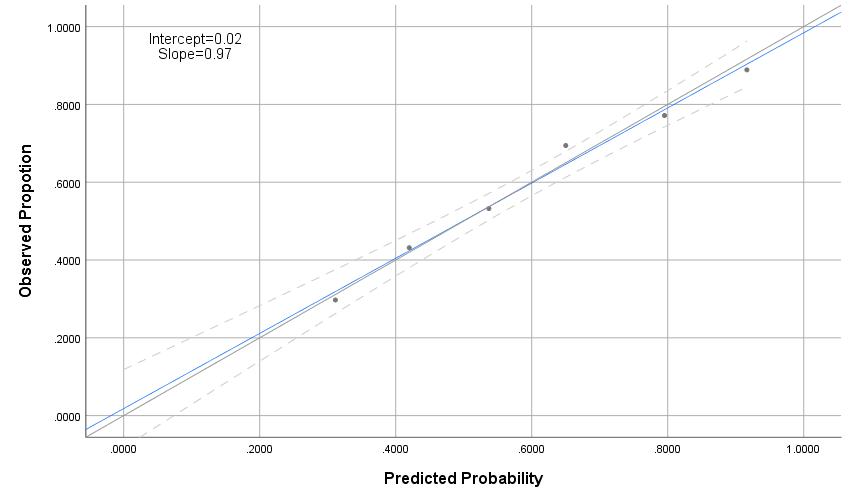

Supplement: Supplementary Figure 2 — The calibration plot of the training sample set. [file Image_2.jpeg]

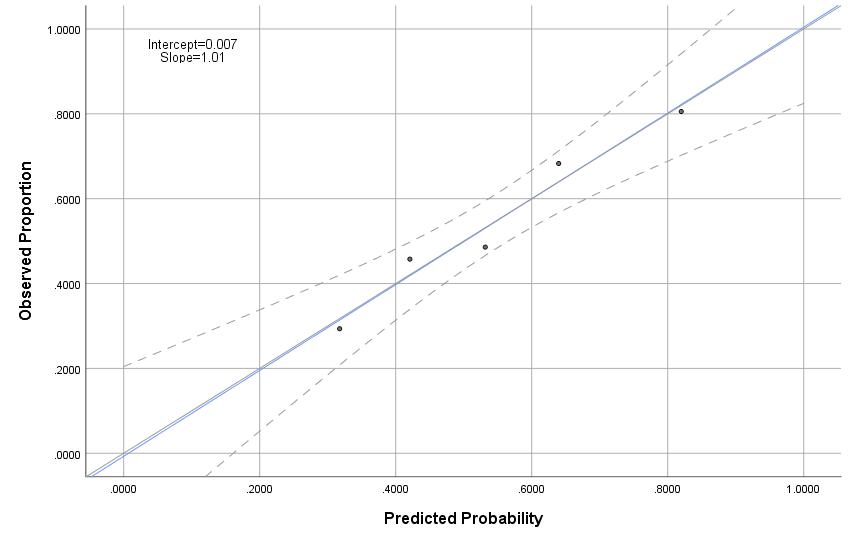

Supplement: Supplementary Figure 3 — The calibration plot of the internal validation sample set. [file Image_3.jpeg]

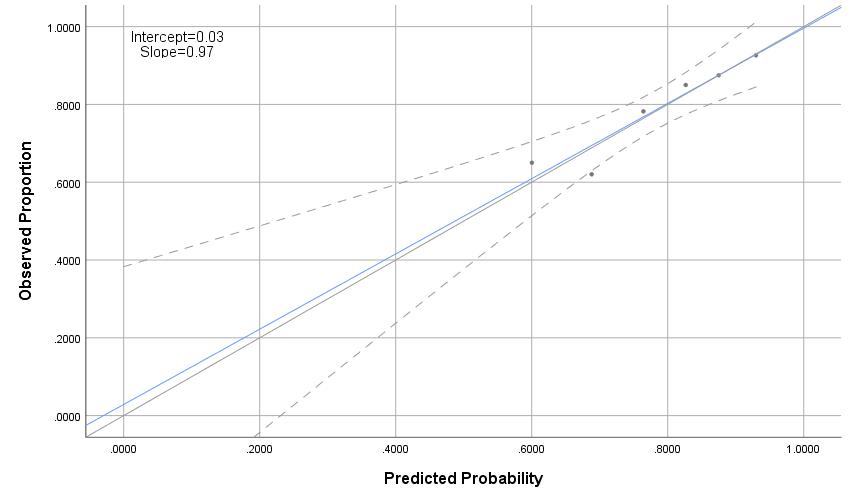

Supplement: Supplementary Figure 4 — The calibration plot of the independent sample set. [file Image_4.jpeg]
